# Spatio-temporal patterns of leptospirosis in Thailand: is flooding a risk factor?

**DOI:** 10.1017/S0950268815000205

**Published:** 2015-03-17

**Authors:** S. SUWANPAKDEE, J. KAEWKUNGWAL, L. J. WHITE, N. ASENSIO, P. RATANAKORN, P. SINGHASIVANON, N. P. J. DAY, W. PAN-NGUM

**Affiliations:** 1 Department of Tropical Hygiene, Faculty of Tropical Medicine, Mahidol University, Thailand; 2 The Monitoring and Surveillance Center for Zoonotic Diseases in Wildlife and Exotic Animals, Faculty of Veterinary Science, Mahidol University, Thailand; 3 Mahidol-Oxford Tropical Medicine Research Unit, Faculty of Tropical Medicine, Mahidol University, Thailand; 4 Faculty of Environment and Resource Studies, Mahidol University, Thailand

**Keywords:** Flooding, human leptospirosis, Thailand

## Abstract

We studied the temporal and spatial patterns of leptospirosis, its association with flooding and animal census data in Thailand. Flood data from 2010 to 2012 were extracted from spatial information taken from satellite images. The incidence rate ratio (IRR) was used to determine the relationship between spatio-temporal flooding patterns and the number of human leptospirosis cases. In addition, the area of flood coverage, duration of waterlogging, time lags between flood events, and a number of potential animal reservoirs were considered in a sub-analysis. There was no significant temporal trend of leptospirosis over the study period. Statistical analysis showed an inconsistent relationship between IRR and flooding across years and regions. Spatially, leptospirosis occurred repeatedly and predominantly in northeastern Thailand. Our findings suggest that flooding is less influential in leptospirosis transmission than previously assumed. High incidence of the disease in the northeastern region is explained by the fact that agriculture and animal farming are important economic activities in this area. The periodic rise and fall of reported leptospirosis cases over time might be explained by seasonal exposure from rice farming activities performed during the rainy season when flood events often occur. We conclude that leptospirosis remains an occupational disease in Thailand.

## INTRODUCTION

Leptospirosis is a globally distributed zoonotic disease caused by a spirochaete bacterium (*Leptospira* spp.). *Leptospira* can enter the human body via abrasions or cuts in the skin, the conjunctiva and intact skin after prolonged immersion in contaminated water and soil. Direct contact with an infected animal's urine and drinking contaminated water are also possible transmission routes [1]. The pathogen colonizes the renal tubule of reservoir hosts (e.g. cattle, buffalo, pigs, dogs, rodents and some wildlife species) and is excreted into the environment in urine [1, 2]. Survival of *Leptospira* in the environment varies markedly from a few weeks to almost a year, depending on its ability to adapt to the environmental conditions [3–5]. In addition, some species such as *L. alstonii* can only adapt to the environment for a short period and lose their virulent phenotype when staying outside the mammalian host [4]. Leptospiral infection often has minimal or no clinical manifestations [6]. The clinical features of symptomatic infections in humans vary from mild to severe. Patients with anicteric leptospirosis present with a febrile illness of sudden onset. Symptoms include chills, myalgia, conjunctival suffusion and abdominal pain. Icteric leptospirosis, the most severe form, often presents with jaundice and possible multiple organ failure [1]. On average, the percentage of confirmed leptospirosis cases representing the severe form ranges from 5% to 10% and the case-fatality rate varies from 1% to 30% [7, 8].

It has been hypothesized that flooding is associated with outbreaks of human leptospirosis because it transiently increases animal–human contacts as animals move from their habitat to human residential areas during periods of flooding. Flooding can also increase the risk of human exposure to the pathogen through contact with contaminated water [9]. Numerous outbreaks of leptospirosis have been reported following flood events in geographically diverse areas of the world [7, 9]. For example, 19·2% of people in an Indian village who presented with symptoms consistent with leptospirosis had positive serological test results after the Orissa cyclone in 1999 [10]. The emergence of leptospirosis followed heavy rainfall and severe flooding in Guyana in 2005 [11] and a leptospirosis outbreak followed a typhoon that caused severe flooding in the Philippines in 2009 [12].

In Thailand, leptospirosis was first observed and reported in 1942 [13]. The annual number of cases in the country has increased markedly since 1996 and a massive outbreak of 14 285 cases was reported in 2000 [14]. A single ecologically successful pathogenic clone of *L. interrogans* serovar Autumnalis, predominant in the rodent population, was associated with this outbreak [15]. For the last 10 years the number of reported cases has stabilized at around 2800–5500 cases per year [8]. The severe flooding during May 2011 to January 2012 affected 65 out of the 77 provinces in Thailand. As a consequence, concerns regarding post-flood leptospirosis outbreaks were raised and thus leptospirosis has since been included as one of the seven disease targets of national surveillance during and after flood events. Interestingly, the annual number of reported leptospirosis cases in 2011 was not significantly different from the previous 5 years [8, 16].

The aim of this study was to assess the influence of flooding on human leptospirosis in a Thai setting. To do this, various estimates of flooding in terms of scale and coverage were used to assess whether flood categorization had an impact on the results. In addition, agricultural animals as potential leptospirosis reservoirs were considered in the analysis on flood associations with human leptospirosis.

## METHODS

### Flood data

Flooding was explored in all 926 districts of Thailand. Flood data from 2010 to 2012 were obtained from spatial information of flooding provided by the Geo-Informatics and Space Technology Development Agency (GISTDA) and subjected to spatial analysis. This process involved georeferencing, classifying the boundaries of flooded areas by colour shading and calculating the percentage of flooded areas [17, 18]. This information was available for areas of the country where extreme flooding was reported. Optical images from many satellites, including RADARSAT-1, RADARSAT-2, COSMO-SkyMed-4 and THEOS, were used to estimate areas of flooding [19]. Flooding was estimated at the district level in three ways. First, we recorded the binary outcome (yes/no flooding) if any part of a district had received flooding. Second, as the actual flooded area could be identified from the colour shading in the flooding images, the flooding coverage (%) per district was calculated from total flooded area of a district divided by total area of that district. Third, the various colour shading of flooded areas taken from the spatial information were further used to categorize the waterlogged areas into three groups, i.e. no waterlogging, <1 week, and ⩾1 week. Then the size of the waterlogged area was estimated from total waterlogged area of a district divided by total area of that district.

### Human leptospirosis data

The monthly number of human leptospirosis case reports by district was collected from 506 surveillance reports made by the Bureau of Epidemiology (BOE), Department of Disease Control, Ministry of Public Health [16]. These reports were generated by the Thai health care system, which is composed of primary-care units, clinics, secondary and tertiary hospitals. Leptospirosis cases were reported weekly to the BOE. Leptospirosis cases were classified into suspected and confirmed cases. The suspected cases included patients who had a history and clinical criteria consistent with leptospirosis. The confirmed cases were defined as those with a positive result of any screening test [latex agglutination, microcapsule agglutination test (MCAT), and lateral flow] or rapid diagnostic test or one of the following tests: microscopic agglutination test (MAT), indirect immunofluorescence assay (IFA), enzyme-linked immunosorbent assay (ELISA), polymerase chain reaction (PCR) or culture [16]. The spatial distribution of reported cases was analysed and represented using hotspot analysis. A GiZ score [20] is mainly used to classify hot and cold spot areas. A hotspot represents an intense clustering of a high number of cases while a cold spot represents a low number of cases.

### Animal census data

Animal species previously shown to be leptospirosis reservoirs, including cattle, buffaloes, pigs and dogs were taken into account for the analysis. This information was provided by the Department of Livestock Development of Thailand at the district level for the period 2010–2012. The association between animal census and human leptospirosis was investigated in flood/non-flood areas during the study period.

### Statistical analysis

Epidemiological data of human leptospirosis cases per district between 2010 and 2012 were organized by year and by flooding period at the country and regional levels. The distribution of leptospirosis cases in the population was assumed to follow a Poisson distribution. To account for over-dispersion of data, negative binomial regression models were used to assess the trend and the association between the monthly number of human leptospirosis cases and flooding by calculating the incidence rate ratio (IRR) [21] and the 95% confidence interval (CI). We also investigated how flooding area coverage, waterlogging period (<1 week, ⩾1 week) and the time lag (1–2 months) between flooding and disease incidence affected leptospirosis cases using a univariate analysis and *Z* test.

In addition to the analysis at the regional scale, a sub-analysis looking at the association of flooding and human cases by low/high incidence areas was also performed. All districts were first classified into high- or low-incidence areas (using the average incidence over the 3-year period as the cut-off), then as previously described, the regression model was used to estimate the adjusted IRR and 95% CI. A multivariate analysis and stepwise selection were performed to study the influence of flooding on leptospirosis occurrence adjusted for the importance of any potential animal reservoirs. Multicollinearity among the independent variables was assessed by observing the size of the standard errors and by calculating the variance inflation factor (VIF) [22].

All statistical analyses were performed using Stata v. 11.0 software (Stata Corp., USA). Spatial analyses were performed using ArcGIS 9.3 software (Environmental Systems Research Institute, USA).

## RESULTS

### Flooding and leptospirosis occurrence

**Fig. 1.**
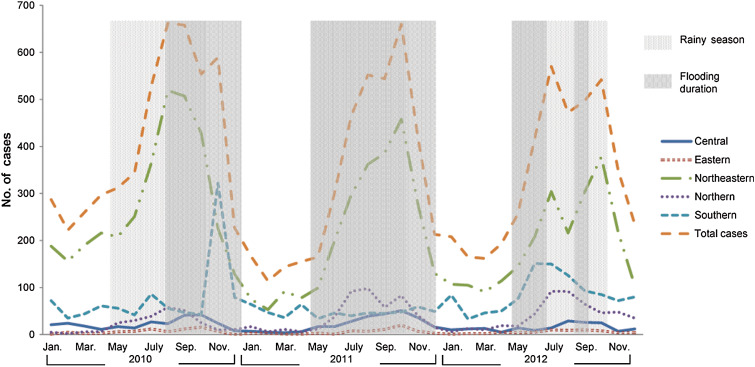
Total human leptospirosis cases in Thailand and by region during 2010 and 2012. Light and dark shaded areas show the rainy season (May–October) and flooding periods, respectively.

Flooding varied across study years, but it always overlapped with the rainy season, i.e. between May and October [23] (Table 1, Fig. 1). Several flooding situations were highlighted during the study period. Towards the end of 2010 severe flooding was reported in the southern regions. In 2011, Thailand was also heavily affected by severe flooding when 65 out of 77 provinces were affected. Further, the duration of this severe flooding covered an unusually long 8-month period. In 2012, the country was relatively dry and drought was a problem in many areas.

**Table 1.** Flooding characteristics, total cases, number of cases and monthly incidence of human leptospirosis at the regional level during flooding 2010–2012 in Thailand

Year	2010			2011			2012		
**Flooding characteristics**									
Months of flooding period*	August–October†			May–December			May–June, September		
Number of flooded districts (%)	454 (49·03)			505 (54·54)			365 (39·42)		
**Leptospirosis case report**									
		During flood period			During flood period			During flood period	
Region	Total	No. cases (% total cases)	Monthly incidence (per 10^5^)	Total	No. cases (% total cases)	Monthly incidence (per 10^5^)	Total	No. cases (% total cases)	Monthly incidence (per 10^5^)
Central	270	106 (39·26)	0·16	270	248 (91·85)	0·14	176	92 (52·27)	0·08
Eastern	77	34 (44·16)	0·28	68	60 (88·24)	0·19	64	38 (59·38)	0·19
Northeastern	3382	1454 (42·99)	2·24	2507	2209 (88·11)	1·28	2294	1176 (51·26)	1·09
Northern	268	134 (50·00)	0·71	476	436 (91·60)	0·87	488	311 (63·73)	0·99
Southern	943	401 (42·52)	2·38	578	367 (63·49)	0·55	1046	596 (56·98)	1·42
Overall	4940	2129 (43·10)	n.a.	3899	3320 (85·15)	n.a.	4068	2213 (54·40)	n.a.

n.a., Not applicable.

* Data from Geo-Informatics and Space Technology Development Agency (Public Agency) [17, 18].

† Except in the southern area where the flood period was November–December.

At the country level, about 43%, 85% and 54% of leptospirosis cases occurred during flooding in years 2010, 2011 and 2012, respectively. On average the proportion of cases per district that occurred during flooding varied significantly from year to year (*P* < 0·01). The proportion of cases that occurred during the 2011 flooding was markedly high (overall 85·15%), but because the duration of flooding was extremely long (i.e. 8 months), the monthly incidence was relatively low in each region that year. Cases occurred predominantly in the northeastern part of the country with about 56–68% of total cases, followed by the southern, northern, central and eastern parts of the country (Table 1).

The overall occurrence of leptospirosis showed a seasonal pattern with a peak in both the rainy season and flood periods (Fig. 1). Trend analysis was applied to monthly leptospirosis cases between 2010 and 2012 using a negative binomial regression. Overall, there was no significant trend in the occurrence of disease over the study period (*P* = 0·86) or by region (*P* = 0·20–0·69), except in the northern part of the country, which showed an increasing trend in human leptospirosis cases (IRR 1·04, *P* < 0·05). By observation, leptospirosis cases seem to be associated with flooding (Fig. 1); however, statistical analysis was used to further investigate such spatio-temporal association.

Table 2 shows the association between flooding (yes/no) and leptospirosis occurrence. The overall IRR varied significantly from year to year, from being a high-risk factor in 2010 and 2011 (IRR 4·03 and 1·65, *P* < 0·01), to being a protective factor in 2012 (IRR 0·66, *P* < 0·01). This overall pattern was the same in those regions where leptospirosis occurrence was relatively high, i.e. in the northeastern and southern regions. In the eastern and central regions where the incidence was relatively low, flooding was consistently present as a protective factor in the eastern region and as a risk factor in the central regions.

**Table 2.** Incidence rate ratio (IRR), 95% confidence interval (CI) and P values of human leptospirosis by region compared to no flooding

		2010		2011		2012	
Factor	Level	IRR (95% CI)	*P* value	IRR (95% CI)	*P* value	IRR (95% CI)	*P* value
Flooding	Overall	4·03 (3·04–5·35)	<0·01	1·65 (1·31–2·07)	<0·01	0·66 (0·50–0·88)	<0·01
	Central	2·01 (1·03–3·92)	0·04	2·26 (1·26–4·03)	<0·01	1·15 (0·52–2·54)	0·74
	Eastern	0·29 (0·11–0·74)	0·01	0·31 (0·12–0·82)	0·02	0·18 (0·04–0·82)	0·03
	Northeastern	4·47 (3·12–6·40)	<0·01	1·65 (1·19–2·31)	<0·01	1·17 (0·80–1·72)	0·41
	Northern	0·56 (0·25–1·24)	0·16	2·01 (1·26–3·20)	<0·01	0·79 (0·31–2·01)	0·62
	Southern	6·64 (3·27–13·53)	<0·01	1·08 (0·61–1·92)	0·78	0·56 (0·28–1·12)	0·10
Flooding with 1-month lag	Overall	3·34 (2·53–4·39)	<0·01	1·66 (1·31–2·08)	<0·01	0·60 (0·45–0·80)	<0·01
Flooding with 2-month lag	Overall	3·52 (2·64–4·69)	<0·01	1·68 (1·33–2·12)	<0·01	0·55 (0·41–0·73)	<0·01
Flooding coverage							
Level = 1 (up to 25%)	Overall	4·71 (3·51–6·33)	<0·01	2·10 (1·66–2·66)	<0·01	0·71 (0·53–0·96)	0·02
Level = 2 (between 25–50%)	Overall	1·72 (1·02–2·89)	0·04	1·25 (0·83–1·90)	0·29	0·21 (0·08–0·59)	<0·01
Level = 3 (above 50%)	Overall	1·02 (0·38–2·72)	0·97	0·65 (0·29–1·43)	0·28	No case report	n.a.
Area size of waterlogging (<1 week)	Overall	1·01 (0·99–1·04)	0·38	n.a.	n.a.	n.a.	n.a.
Area size of waterlogging (⩾1 week)	Overall	1·01 (0·99–1·04)	0·39	n.a.	n.a.	n.a.	n.a.

n.a., Not available.

The IRR with time lag (1 or 2 months after flooding) for the overall districts showed the same pattern of relationship between flooding and disease occurrence as the IRR without the time lag. However, with the time lag, the IRR for all three years represented a weak association compared to when the time lag was not included in the analysis. We also tested time lags of 1, 2 and 3 weeks and got results consistent with 1- or 2-month time lags (data not shown).

Since the time lag did not improve the model fit, proving to be the least important variable in this case, it was omitted in further analyses.

The IRR of flooding coverage, when categorized into four levels (0, no flood; 1, ⩽25%; 2, 25–50%, 3, >50%), showed a similar pattern to when flooding was considered as a binary factor (yes/no). The percentage of flooding coverage was not significantly associated with the incidence of disease. By contrast, flooding coverage was negatively correlated with disease incidence, i.e. areas with high flooding coverage tended to have low incidence of leptospirosis.

Last, the IRR showed no significant association with either short-term or long-term waterlogging periods (IRR 1·01, *P* = 0·38 and 0·39). In conclusion, the analysis indicates that flooding simply characterized in binary form (yes/no) best describes the association between flooding and the occurrence of human leptospirosis.

**Fig. 2.**
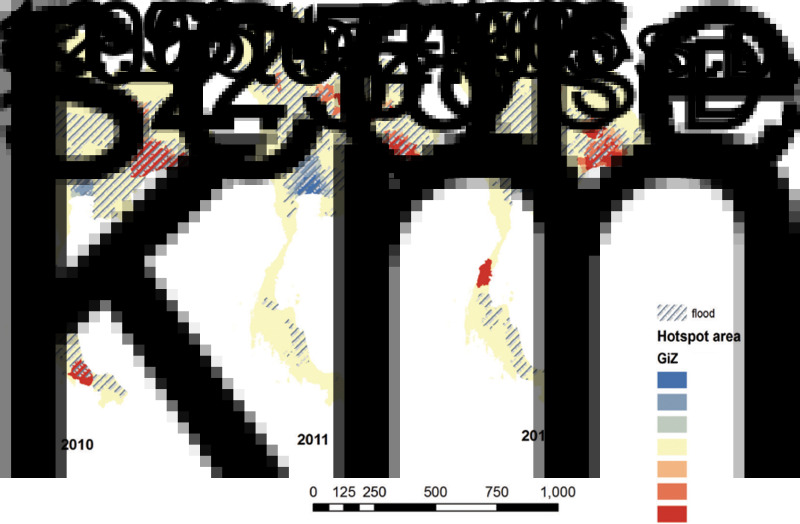
Hotspot analysis of human leptospirosis cases and flooding between 2010 and 2012, Thailand.

The spatial distribution of leptospirosis incidence in the country during the study period is shown in Fig. 2 using hotspot analysis. The flooding situation was most severe in 2011 when flooding area coverage was the largest in the three years. In 2012, there were fewer reports of flooding and even drought events occurred. Leptospirosis cases occurred repeatedly in the same areas over the study periods, in particular the northeastern region. The geographical distribution of disease and the hotspot analysis lend support to the statistical analysis, which indicates that the association between flooding and human leptospirosis is not a direct effect due to some potential confounding factors which need further investigation including, for example, seasonal rice farming and level of contacts with potential animal reservoirs such as buffaloes.

Following on from the hotspot analysis, a sub-analysis was performed by observing the association between flooding and human cases by low-/high-incidence areas. The cut-off incidence used to classified a ‘low-’ and ‘high’-risk area was the average incidence over the study period, i.e. 6·8 cases/100 000 population. The IRRs of flooding after adjusting for the incidence level were estimated through the regression model. Similar results were found to those at the country level, i.e. the IRRs were greater in the first year of the study even though flooding was overall more severe in the second year (IRR 2·31 and 1·64). In the last year of the study, flooding was presented as a protective factor (IRR 0·87); however, this was not statistically significant (*P* = 0·25).

Regarding a possible association between the district incidence of leptospirosis and the number of potential animal reservoirs, almost all animal species were positively associated with the number of human cases in all years of study based on univariate analysis. However, by multivariate analysis, when both flooding and animal data were considered together, only flooding and the number of buffaloes were consistently associated with the number of cases. By using stepwise selection, only flooding and number of buffaloes would remain in the final model (results not shown). The impact of flooding on the number of human leptospirosis cases after adjusting for the number of buffaloes was slightly reduced in each year (Table 3). Both the small values of the standard errors for all model coefficients and the VIF indicated no concern for the multicollinearity issue in the regression models (VIF <2).

**Table 3.** Incidence rate ratio (IRR) and 95% confidence interval (CI) of human leptospirosis adjusted for flooding and animal data in the univariate and final multivariate models

	IRR (95% CI, *P* value)					
	2010		2011		2012	
Factor	Univariate	Multivariate	Univariate	Multivariate	Univariate	Multivariate
Flooding	4·03 (3·04–5·35, < 0·01)	3·19 (2·39–4·25, <0·01)	1·65 (1·31–2·07, <0·01)	1·13 (0·89–1·44, 0·31)	0·66 (0·50–0·88, <0·01)	0·58 (0·43–0·79, <0·01)
log_10_ (no. of buffalo)	2·17 (1·84–2·56, <0·01)	1·93 (1·59–2·36, <0·01)	2·16 (1·89–2·46, <0·01)	1·94 (1·64–2·29, <0·01)	1·65 (1·40–1·93, <0·01)	1·78 (1·45–2·18, <0·01)
log_10_ (no. of cows)	3·83 (2·86–5·12, <0·01)	1·15 (0·76–1·74, 0·50)	2·82 (2·30–3·45, <0·01)	1·31 (0·96–1·78, 0·09)	1·76 (1·36–2·27, <0·01)	1·03 (0·71–1·50, 0·87)
log_10_ (no. of pigs)	1·75 (1·32–2·33, <0·01)	1·10 (0·81–1·50, 0·53)	1·39 (1·14–1·70, <0·01)	0·98 (0·78–1·23, 0·88)	1·19 (0·92–1·54, 0·18)	1·14 (0·84–1·54, 0·39)
log_10_ (no. of dogs)	2·77 (2·00–3·84, <0·01)	1·04 (0·63–1·70, 0·88)	2·36 (1·87–2·97, <0·01)	0·93 (0·63–1·39, 0·74)	1·51 (1·07–2·12, 0·02)	0·61 (0·36–1·04, 0·07)

## DISCUSSION

Numerous studies assessing the relationship between leptospirosis and flooding have been conducted directly after flooding [9, 24, 25]. Therefore, an increased number of cases after flooding have been used as evidence of disease outbreaks as a result of flooding. However, such studies may not have considered a sufficient spatio-temporal scale to support such a conclusion. Although our findings show that flooding is associated with the number of human leptospirosis cases, the pattern and the strength of this association were not consistent. Flooding was a strong risk factor for leptospirosis in 2010 and 2011 while it was a protective factor in 2012. Moreover, such association diminisherd after adjustment for the number of buffaloes in the final regression model. Other quantitative measures of flooding including duration of waterlogging and flooding coverage were less associated with disease incidence compared to the binary estimate of flooding. These results together with the spatial distribution of leptospirosis cases provide evidence that flood events are not directly correlated with the occurrence of human leptospirosis.

Leptospirosis cases predominantly occurred in the northeastern and southern parts of Thailand where the majority of the population is involved in agricultural work. The high risk of leptospirosis infection may come from the fact that on average farmers spend several hours on wet and muddy land working in proximity to potential reservoir hosts such as rodents, cattle, and domestic pets. Indeed, leptospirosis in Thailand is prevalent mainly in farmers and their employees [8], and case reports linking Thai rice workers with leptospirosis in northeastern Thailand have been reported [26, 27]. Many farming activities take place during the rainy season often temporally overlapping with flooding and leptospirosis cases, which may create the false impression of a direct association between flooding and disease. Unrelated to flooding, suitable ecological conditions for survival of the pathogen [1, 9, 28] may influence the incidence of human leptospirosis in this region.

After the severe flooding in 2011, a post-flooding study from Bangkok metropolitan region rarely found pathogenic *Leptospira* in floodwater samples, and the number of human leptospirosis cases reported was relatively low [29]. In addition to the ecological reason mentioned above, other reasons relating to both host and pathogen may exist to explain the low prevalence of human leptospirosis cases following severe flooding. In humans, the evacuation of people living in flooded areas, good personal hygiene and personal protective equipment to avoid human–pathogen contact or socioeconomic differences may have made flood-affected individuals less likely to become infected, for example. Regarding the pathogen, large volumes of water during flooding may have led to a dilution effect, reducing *Leptospira* levels below that of the infective dose, while variation in different strains' virulence by region may have resulted in fewer reported cases in some flooded areas. Further field data and laboratory investigations are required to test these hypotheses.

The buffalo was the only animal identified by the regression model to be associated with human leptospirosis in this study. The presence of buffaloes may be seen as an indicator of human leptospirosis in the area although currently the use of buffaloes in agricultural activities is minimal and mostly occurs among retail farmers in rural settings. Buffalo husbandry is a sociocultural activity where buffaloes represent wealth, with meat being the main product [30]. Humans may be exposed to the pathogen in daily farming activities, such as moving animals to grazing land or cleaning their enclosures.

Interestingly, previous leptospirosis seroprevalence studies found about 30–60% seropositivity in buffaloes compared to much lower scores for other possible animal reservoirs in Thailand [31, 32]. The serovars found in buffaloes, such as Shermani, Pomona, Sejroe, Bratislava and Bataviae [31–33], can be pathogenic in humans. Furthermore, serovars Sejroe and Pomona can cause acute leptospirosis in humans [34]. The serovars Bratislava and Sejroe, linked with most human cases, have been found in buffaloes in Nakhon Ratchasima province, Thailand [33]. This evidence suggests that the buffalo could play a role as an important animal reservoir of leptospirosis in Thailand. More evidence-based studies are required before claiming buffaloes as a risk factor for human leptospirosis.

The strength of our study over previous studies is the use of spatial flooding information over a period of several years, as it includes various estimations of flooding in terms of scale and coverage. Moreover, data were collected independently of disease occurrence through the study period to minimize bias which may have occurred as a result of over-searching of cases in a post-flood investigation. However, some potential limitations of this study should be noted: (*a*) the lack of information on other important animal reservoir hosts such as rodents; (*b*) some leptospirosis cases potentially being under-recognized or under-reported due to difficulties in differentiating leptospirosis from other febrile illnesses (e.g. physicians' subjective diagnosis and lack of experience with the disease, non-specific screening tests, or very mild symptoms which may not have been diagnosed as leptospirosis) [1, 8, 9]; and finally (*c*) uncertainty on the precise timing of flood events during some periods because the acquisition of satellite images depends both on the orbit of the satellite as well as the time of picture capture. This, therefore, resulted in discontinuity of images temporally and spatially [19]. The latter point reduced the power of the analysis as the association of cases and flooding cannot be considered per flood event but as a whole period of floods reported (see Table 1). Note that floods, in this study, were generally defined as those that occurred mostly due to heavy rain during the rainy season. The findings may not be comparable to other regions in the world where flooding may occur differently, e.g. ecological mechanisms would be distinct in flash-floods caused by tropical storms.

Further investigations should collect and incorporate information about the human host in the analysis, including human behaviour, particularly personal hygiene and human–animal–environment contact patterns. A better understanding of leptospirosis and its risk factors will enable the design of optimal disease prevention and control strategies.
